# Fatty Acid Solubilizer from the Oral Disk of the Blowfly

**DOI:** 10.1371/journal.pone.0051779

**Published:** 2013-01-11

**Authors:** Yuko Ishida, Jun Ishibashi, Walter S. Leal

**Affiliations:** 1 Department of Biology, Graduate School of Science, Kobe University, Nada, Kobe, Hyogo, Japan; 2 National Institute of Agrobiological Sciences, Tsukuba, Ibaraki, Japan; 3 Department of Entomology, University of California Davis, Davis, California, United States of America; New Mexico State University, United States of America

## Abstract

**Background:**

Blowflies are economic pests of the wool industry and potential vectors for epidemics. The establishment of a pesticide-free, environmentally friendly blowfly control strategy is necessary. Blowflies must feed on meat in order to initiate the cascade of events that are involved in reproduction including juvenile hormone synthesis, vitellogenesis, and mating. During feeding blowflies regurgitate salivary lipase, which may play a role in releasing fatty acids from triglycerides that are found in food. However, long-chain fatty acids show low solubility in aqueous solutions. In order to solubilize and ingest the released hydrophobic fatty acids, the blowflies must use a solubilizer.

**Methodology:**

We applied native PAGE, Edman degradation, cDNA cloning, and RT-PCR to characterize a protein that accumulated in the oral disk of the black blowfly, *Phormia regina*. *In situ* hybridization was carried out to localize the expression at the cellular level. A fluorescence competitive binding assay was used to identify potential ligands of this protein.

**Conclusion:**

A protein newly identified from *P. regina* (PregOBP56a) belonged to the classic odorant-binding protein (OBP) family. This gene was expressed in a cluster of cells that was localized between pseudotracheae on the oral disk, which are not accessory cells of the taste peg chemosensory sensilla that normally synthesize OBPs. At pH 7 and pH 6, PregOBP56a bound palmitic, stearic, oleic, and linoleic acids, that are mainly found in chicken meat. The binding affinity of PregOBP56a decreased at pH 5. We propose that PregOBP56a is a protein that solubilizes fatty acids during feeding and subsequently helps to deliver the fatty acids to the midgut where it may help in the process of reproduction. As such, PregOBP56a is a potential molecular target for controlling the blowfly.

## Introduction

In the aftermath of the earthquake and subsequent tsunami that occurred in northern Japan on March 11, 2011, outbreaks of blowflies emerged from rotting fish, and affected public health [1]. Typically, the larvae of blowfly species produce wound myiasis. When blowflies strike sheep, the result is a reduction in the quality and quantity of wool, ewe infertility, and death, resulting in considerable economical losses in the wool industries [2], [3]. Blowflies are also considered potential vectors of H5N1 avian influenza virus by mechanical transmission [4]. Additionally, there are problems with an increased incidence of insecticide-resistant blowflies and insecticide residues in the products of livestock [5]. Therefore, the establishment of environmentally friendly strategies for the control of blowflies is necessary especially in the wool industry and in terms of public health.

Blowflies must feed on meat in order to initiate the events involved in reproduction. In the black blowfly, *Phormia regina*, juvenile hormone (JH) biosynthesis is activated through the mevalonate pathway within 12 hours of feeding [6], [7], [8], [9]. The increase in JH titer subsequently turns on male mating behavior [10]. In females, the increase in JH titer induces lipid and protein accumulation in the fat body from the digested meal, and increases vitellogenin synthesis. The vitellogenin and lipids released into hemolymph from the fat body accumulate into the developing oocytes under control of JH. JH is also involved in the regulation of male acceptance by females [8], [11], [12]. These findings imply that the newly ingested fatty acids are utilized immediately for JH synthesis in the corpora allata and continuously for vitellogenin synthesis in the fat body.

During feeding blowflies regurgitate salivary lipase, which might play a role in releasing fatty acids from triglycerides [13], [14]. However, the solubility of long-chain fatty acids is extremely low in aqueous solution [15]. Although its exact function remains elusive, fatty acid-binding protein 5 has been identified in the human saliva which may help to solubilize long-chain fatty acids in human [16]. In order to effectively solubilize and ingest the hydrophobic fatty acids, the blowfly likely uses a similar solubilizer.

In order to identify fatty acid-binding protein(s) from the black blowfly, *P. regina*, we searched here protein expression from various tissues, including the oral disk, which makes direct contacts with the meal and presumably can accumulate proteins of our interest. A protein that accumulated at relatively high levels was detected specifically in the oral disk. cDNA cloning and phylogenetic analysis demonstrated that the protein belongs to the insect classic odorant-binding protein (OBP) family, which plays a role in solubilizing hydrophobic ligands in aqueous solution [17], [18], [19]. We further characterized this protein by RT-PCR, *in situ* hybridization, and a fluorescence competitive binding assay, and discuss its presumed function.

## Materials and Methods

### Protein identification

A colony of the black blowfly, *P. regina* was maintained under the conditions described by Nishimura et al. [20]. In order to identify an oral disk-specific protein, each experimental tissue at day 3 of the adult stage was collected using fine clean forceps under a microscope. The tissue was homogenized in native PAGE sample buffer using a disposable pellet pestle (Trefflab, Degersheim, Switzerland). The protein homogenate was separated by 15% native PAGE (e-pagel, ATTO, Tokyo, Japan), and the gel was stained with Coomassie Brilliant Blue R-250.

In order to determine the N-terminal amino acid sequence of the oral disk-specific protein, protein was extracted from 500 labella that were collected from the blowflies. The labella were homogenized as described above in 10 mM Tris-HCl, pH 8, and the homogenate was centrifuged at 13,000 rpm at 4°C for 10 min to remove debris. The supernatant was partially separated on a column of DEAE Sepharose Fast Flow (200 µl gel volume, GE Healthcare, Little Chalfont, United Kingdom) by stepwise elution with 10 mM Tris-HCl, pH 8, containing 50, 100, 200, 250, 300, 400, and 500 mM NaCl. Proteins in the fractions containing 150 and 200 mM NaCl were concentrated, and subsequently separated by native PAGE using Tris-glycine running buffer. Following separation, the protein was electrophoretically transferred onto a polyvinylidene fluoride membrane (Millipore, Billerica, MA, USA) using 10 mM CAPS transfer buffer containing 10% methanol, pH 11, using a Trans-Blot SD Semi-Dry Transfer Cell (Bio-Rad Laboratories, K. K. Tokyo, Japan). Protein bands were visualized by Coomassie Brilliant Blue staining. The N-terminal sequences of a newly identified oral disk-specific protein and a chemical sense-related lipophilic ligand-binding protein (CRLBP) were determined by Edman degradation.

### cDNA cloning and bioinformatics analysis

Total RNA was extracted from an oral disk at day 0 adult stage using TRIzol Reagent (Life Technologies, Carlsbad, CA, USA). The total RNA was used as template to synthesize cDNA using a SMART RACE cDNA Amplification Kit (Clontech, Mountain View, CA, USA) and Superscript II (Life Technologies) as a reverse transcriptase. The cDNA was treated with RNase H (New England Biolabs, Ipswich, MA, USA).

3′-RACE was performed using the degenerate primer, EEQKAKV-1, 5′-GA(A/G)GA(A/G)CA(A/G)AA(A/G)GC(A/C/G/T)AA(A/G)GT-3′, designed on the basis of N-terminal sequence of an oral disk specific protein, EEQKAKV, which was determined by Edman degradation, and UPM supplied in the SMART RACE cDNA Amplification Kit, and *Takara Ex Taq* (Takara Bio, Otsu, Shiga, Japan) as a *Taq* DNA polymerase. The PCR cycles were as follows: 94°C for 3 min, 35 cycles of 94°C for 1 min, 55°C for 1 min, and 72°C for 1 min. The amplified PCR product was gel-purified, ligated into the *Eco* RV site in pBluescript II SK (+) (Agilent Technologies, Santa Clara, CA, USA), and sequenced on an ABI 3130×1 sequencer (Applied Biosystems, Foster, CA, USA). 5′-RACE was performed using the gene-specific primer PregOBP56a-1 (5′-AGATTTTGAAACCGGTATCACAACGATC-3′) and UPM and KOD-plus- (Toyobo, Osaka, Japan) as a *Taq* DNA polymerase. The PCR amplification was performed as follows: 95°C for 2 min, 35 cycles of 94°C for 15 s, 56°C for 30 s, and 68°C for 1 min. In order to further confirm the 3′-region, a second 3′-RACE was performed using the gene-specific primer PregOBP56a-2 (5′-GCCGGTGCATTTGCTCACCTTGAATTGACC-3′) and UPM. PCR was carried out as follows: 95°C for 2 min, 35 cycles of 94°C for 15 s, 67°C for 30 s, and 68°C for 1 min. The full-length cDNA sequence was determined using 9 independent clones to avoid PCR-derived sequence errors.

DNA sequence and blastp searches were performed using CLC DNA Workbench 5.7 software (CLC Bio, Aarhus N, Denmark). Phylogenetic analysis was carried out using the neighbour-joining method with 1000 bootstrap replicates using MEGA5 software [21] after sequence alignment with ClustalW.

### RT-PCR

Total RNA was extracted from each experimental tissue and reverse-transcribed as described above. The PCR program was carried out using primers PregOBP56a-1 and PregOBP56a-2, or CRLBP-1 (5′-GAAGCTGGGGCATCAGATGCCGACTTTG-3′) and CRLBP-2 (5′-GCCATCATCACTCATCACTCCGAATTTC-3′) using KOD-plus- as a *Taq* DNA polymerase. The following cycles were used: 95°C for 2 min, 20 cycles of 94°C for 15 s, 59°C for 30 s, and 68°C for 1 min. The expression of *actin* was detected as an internal control [22]. The PCR-amplified products were separated on a 1.5% agarose gel electrophoresis, visualized under UV using a gel documentation system (Printgraph, ATTO, Tokyo, Japan), and cropped with Photoshop CS3 (Adobe, San Jose, CA, USA).

### *In situ* hybridization

*In situ* hybridization was carried out according to the method described by Vosshall et al. [23]. The PCR amplicon generated by primers PregOBP56a-1 and PregOBP56a-2 was inserted into the *Eco* RV site of pBluescript II SK(+), digested with *Eco* RI and *Hind* III, and ligated into corresponding sites of pSPT18 (Roche Applied Science, Indianapolis, IN, USA). Digoxigenin (DIG)-labeled anti-sense and sense (control) probes were synthesized using a DIG-RNA labeling Kit (SP6/T7) (Roche Applied Science) according to the instruction manual. Labella were collected from flies at day 0 adult stage, embedded in Tissue-TeK O.C.T. Compound (Sakura Finetek Japan, Tokyo, Japan), cut into 15 µm thick slices using a microtome, and dried on amino silane-coated glass slides (Matsunami Glass, Osaka, Japan). After 4% paraformaldehyde fixation for 10 min, the specimens were acetylated, prehybridized in hybridization buffer (50% formamide, 5× SSC, 5× Denhardts, 250 µg/ml yeast tRNA, 500 µg/ml sonicated salmon sperm DNA, 50 µg/ml heparin, 2.5 mM EDTA, 0.1% Tween-20, and 0.25% CHAPS) at 55°C for 1 h, and hybridized overnight with 0.7 µg/ml of probe. Following probe hybridization, the specimen was washed 3 times with 5× SSC at 65°C, 0.2× SSC at 65°C for 20 min, and treated with PBS containing 0.1% Triton X-100 for 10 min. The specimen was then blocked with PBS containing 0.1% Triton X-100 and 10% normal goat serum (Wako Pure Chemical Industries, Osaka, Japan) for 1 h, and then reacted with anti-digoxigenin-Rhodamine Fab fragments (Roche Applied Science) at 1∶1,000 dilution for 3 h. The slices embedded with FluorSave Reagent (Calbiochem, Darmstadt, Germany) were observed on a FLUOVIEW FV1000 confocal laser scanning microscope (Olympus, Tokyo, Japan). Other procedures were performed as reported previously [24].

### Recombinant protein expression, purification, and evaluation

Recombinant PregOBP56a was expressed under oxidative conditions of the periplasmic space of bacteria resulting in the spontaneous formation of disulfide bridges, and purified as described previously [25]. In brief, cDNA encoding mature PregOBP56a was inserted into recognition sites of *Msc* I and *Eco* RI of pET-22b(+) (Novagen, Madison, WI, USA) and the construct was confirmed by DNA sequencing. The BL21(DE3) transformed with the bacterial expression vector was inoculated in LB medium including Carbenicillin as an antibiotic, cultured at 200 rpm, 28°C for 16 h, induced by addition of isopropyl ß-D-1-thiogalactopyranoside for 3 h, and harvested by centrifugation. The recombinant protein was extracted by 3 cycles of freeze and thaw procedure, removed debris by centrifugation, and purified by a combination with ion-exchange chromatography and gel filtration as described previously [26].

The purified recombinant PregOBP56a was dissolved in water and mixed with the matrix solution, saturated sinapinic acid. The recombinant protein/matrix mixture was spotted on a target plate and dried at room temperature. The MALDI TOF mass spectrum was collected in the positive ion reflector mode with an Autoflex III mass spectrometer (Bruker Daltonics, MA, USA). The N-terminal amino acids sequence of the recombinant protein was confirmed by Edman degradation.

Mass-finger print analysis was performed as follows. Five micrograms of the recombinant PregOBP56a (5 µl of a 1 µg/µl solution) was reduced by adding 10 µl of dithiothreitol solution (10 µg/µl in 100 mM Tris-HCl pH 8.5) under a nitrogen atmosphere for 2 hours at 45°C. The reduced protein was then S-carboxymethylated by adding 10 µl of sodium iodoacetate solution (25 µg/µl in 100 mM Tris-HCl pH 8.5) for 1 hour at 45°C. The carboxymethylated recombinant PregOBP56a was purified by reverse phase HPLC (PegasilODS, 4.6×250 mm, Senshu Scientific, Tokyo, Japan), equilibrated with 20% acetonitrile containing 0.05% trifluoroacetate, and eluted with a linear gradient of 20–40% acetonitrile over 20 min at a flow rate of 1.0 ml/min. The purified protein was lyophilized and then resuspended in 10 µl of H_2_O, and 5 µl of this protein solution was trypsinized by the addition of 5 µl of trypsin solution (Promega, Trypsin Gold, Mass Spectrometry Grade, 20 ng/µl in 25 mM ammonium bicarbonate) for 20 hours at 37°C. Subsequently, the trypsinized fragments were separated by MALDI TOF mass spectrometer and the pattern was analyzed with AutoflexIII (Bruker Daltonics) using α-cyano-4-hydroxycinnamic acid as a matrix.

### Circular dichroism analysis

Far UV circular dichroism spectra at a range of 187–260 nm were recorded using a J-810 spectropolarimeter (Jasco, Easton, MD, USA). The purified recombinant protein was diluted to 4 mg/ml in 20 mM ammonium acetate, pH 7 or 20 mM sodium acetate, pH 5.

### Fluorescence competitive binding assay

The fluorescence competitive binding assay was carried out as described previously [27], [28]. In brief, *N*-phenyl-1-naphthylamine (1-NPN) (Tokyo Chemical Industry, Tokyo, Japan) was used as a fluorescent reporter. To measure the affinity of the recombinant PregOBP56a for 1-NPN, a 10 µg/ml solution of PregOBP56a in 20 mM phosphate-citrate buffer at pH 7, 6, or 5 was titrated with aliquots of 3.2 mM ligand solution in ethanol to final concentrations of 1.6–19.2 µM. Fluorescence was measured using a spectrofluorophotometer (F-2000, Hitach Hi-Technologies, Tokyo, Japan) after incubation for 2 min. Each sample, in the 2-ml SQ cuvette, was excited at 337 nm with emission and excitation slit widths of 10 nm and 10 nm. The emission spectra were recorded at a range of 350–500 nm. Cuvettes containing an appropriate concentration of 1-NPN were measured in parallel and the values obtained were subtracted as background fluorescence. The fluorescence intensity (440 nm) of a mixture of 1-NPN and recombinant protein without competitor was used as a reference (100%) to normalize the following measurements. The dissociation constant was calculated using Prism 6 software (GraphPad Software, La Jolla, CA, USA).

Decanoic acid, palmitic acid, stearic acid, oleic acid, linoleic acid, palmityl acetate, and cholesterol (Tokyo Chemical Industry) were used as competitors in the fluorescence competitive binding assay. After incubation of PregOBP56a with 1-NPN at a maximum binding concentration of 16 µM (pH 7), 14.4 µM (pH 6), or 19.2 µM (pH 5), each competitor was added to the PregOBP56a/1-NPN mixture at concentrations of 0-16 µM. The fluorescent intensity (440 nm) of the mixture of 1-NPN and recombinant protein without competitor was used as a reference (100%) to normalize the following measurements. The dissociation constant was calculated using Prism 6 software.

## Results

### Identification and cDNA cloning of an oral disk-specific protein

**Figure 1:**
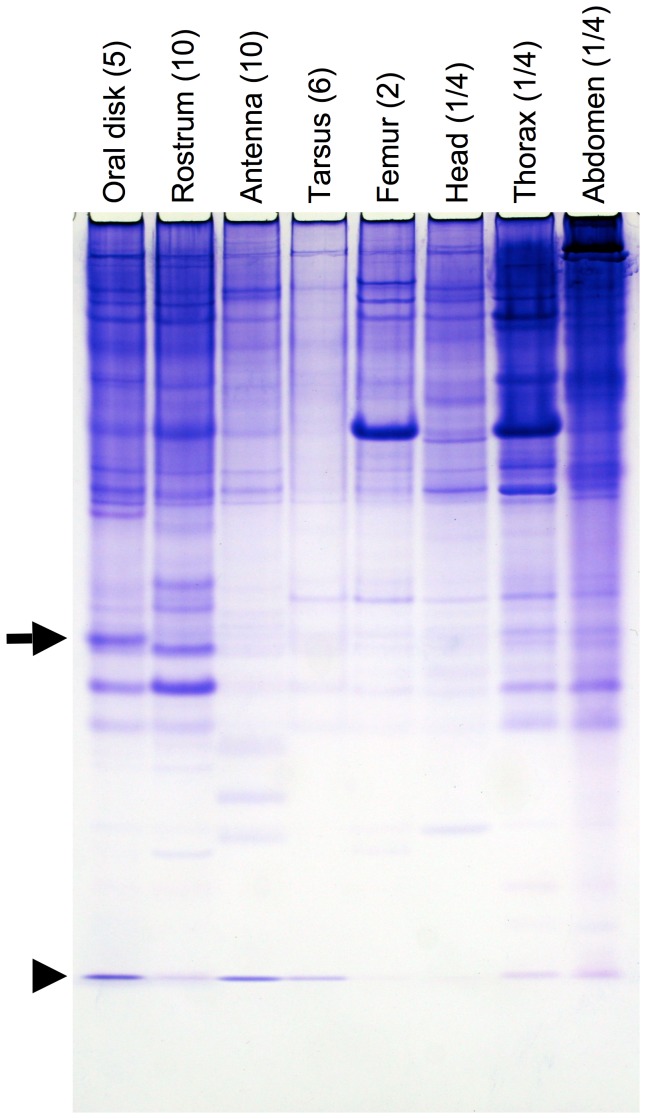
Identification of an oral disk-specific protein by native PAGE analysis. Protein was extracted from each experimental tissue of females at day 3 adult stage, separated by 15% native PAGE, and visualized with Coomassie Brilliant Blue-R250 staining. The source of the protein is shown at top of the gel profile. The number of individuals from which the tissues were collected is indicated in parenthesis. The arrow and arrowhead indicate the migration of an oral disk-specific protein and a chemical sense-related lipophilic ligand-binding protein (CRLBP), respectively.

The aim of this research was to identify a potential solubilizer of long-chain fatty acids that are derived from the diet of the black blowfly, *P. regina*. In order to do this, protein from various tissues including the oral disk of female adult blowflies were analyzed by 15% native PAGE. Two major proteins that migrated to the middle of the native PAGE gel were detected from the oral disk. The slowly migrating protein was only observed in the oral disk (Figure 1, indicated by an arrow), but not in the rostrum. The oral disk-specific protein was identically observed in males (data not shown). It is known that a chemical sense-related lipophilic ligand-binding protein (CRLBP) is predominantly expressed in the oral disk of the blowfly. CRLBP belongs to the classic OBP family and is responsible for taste [29]. In order to characterize the newly identified oral disk-specific protein, Edman degradation was carried out. The N-terminal amino acid sequence of the protein, HLELTEEQKAKV, was different from that of CRLBP, ELTKEEAITIATE. Further experiments of Edman degradation indicated that CRLBP was a protein migrating at front of the native PAGE under our experimental conditions (Tris-glycine running buffer) (Figure 1, indicated by an arrowhead). On the basis of the staining intensity of Coomassie Brilliant Blue, the relative amount of our newly identified oral disk-specific protein was significantly higher than that of an OBP related to chemical reception.

**Figure 2:**
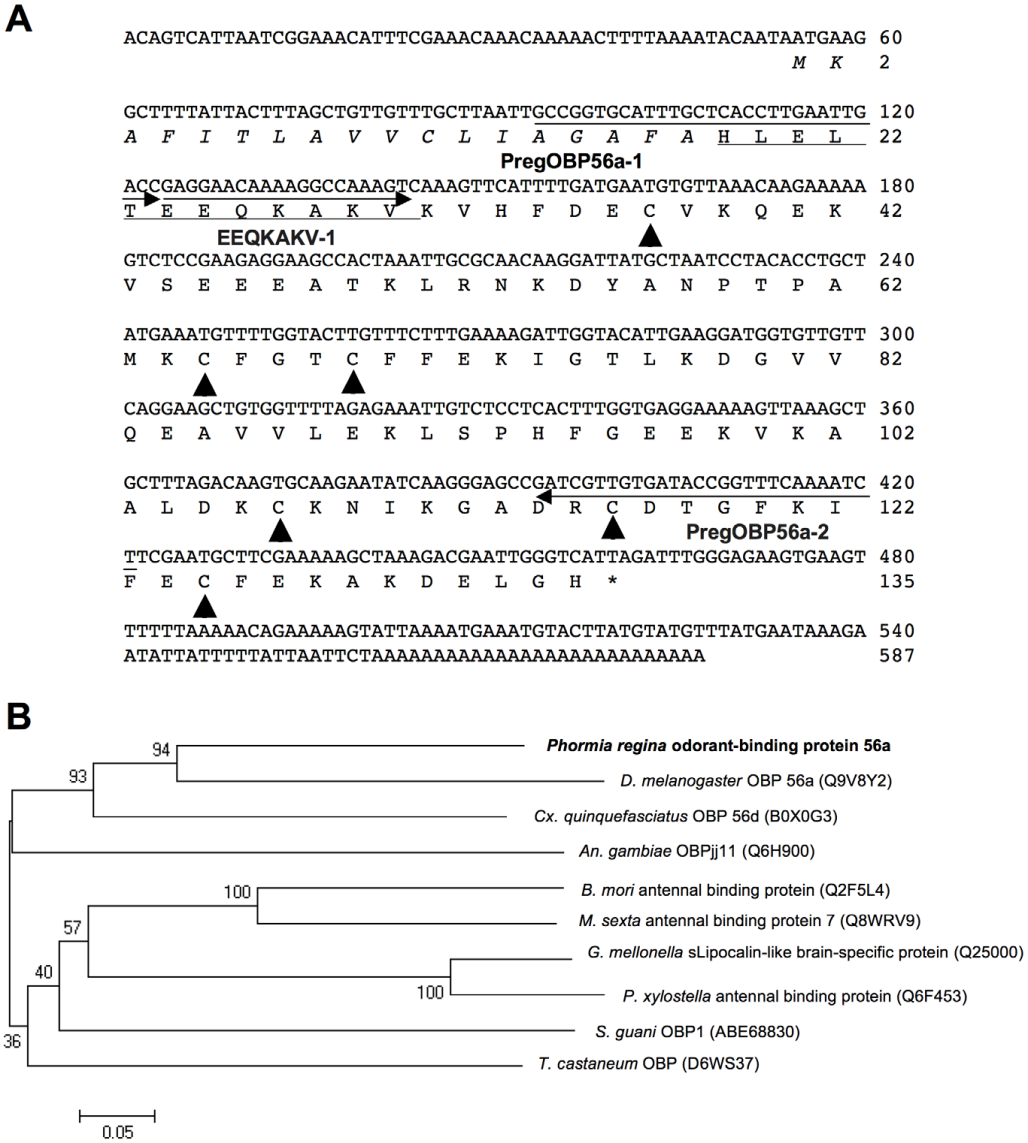
Nucleotide and deduced amino acid sequences and phylogenetic analysis of oral disk-specific protein (PregOBP56a). A, cDNA and deduced amino acid sequences of an oral disk-specific protein. A signal peptide is shown in italic. N-terminal amino acid sequence determined by Edman degradation is underlined. Arrowheads show 6 cysteine residues, which are hallmarks of insect classic OBPs. The three arrows indicate annealing sites of a degenerate (EEQKAKV-1) and two gene-specific (PregOBP56a-1 and PregOBP56a-2) primers, B, Phylogenetic tree of odorant-binding protein 56a like-protein sequences. *Phormia regina* odorant-binding protein 56a is shown in bold. Accession numbers of each protein are given in the parenthesis. Bootstrap values were determined from 1,000 replications. Bar indicates 5% divergence.

By 5′- and 3′-RACE using a degenerate primer designed on the basis of N-terminal sequence, gene-specific primers, and UPM, a cDNA sequence of 587 bp was obtained (accession number, AB697136). The predicted mature protein was composed of 117 amino acid residues including 6 cysteine residues, which are hallmarks of insect classic OBP (Figure 2A). OBP plays a role in solubilizing hydrophobic ligands in aqueous solution in insect chemosensory organs [17], [18], [19]. This protein has a calculated molecular weight of 13,251 Da and isoelectric point of 6.36. Phylogenetic analysis indicated that this protein belonged to the cluster of dipteran OBPs (DmelOBP56a, CquiOBP56d, and AgamOBPjj11) but did not cluster with insect antennal binding proteins from other taxa (Figure 2B). Blastp search showed that this protein had 54% identity with DmelOBP56a (accession number, Q9V8Y2). Thus, we named this oral disk-specific protein, *P. regina* odorant-binding protein 56a, PregOBP56a, although it is unlikely involved in olfaction.

### Localization of *PregOBP56a* expression

**Figure 3:**
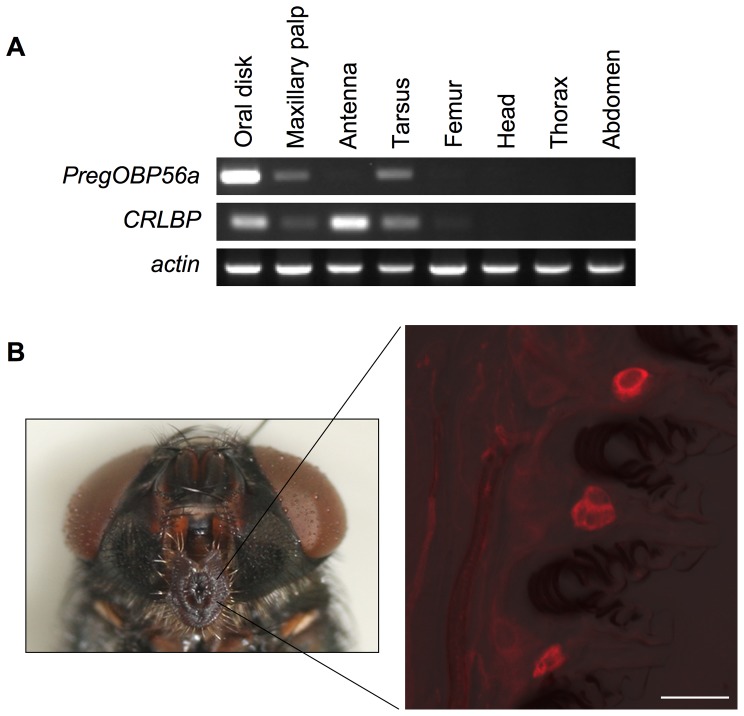
Expression of *PregOBP56a*. A, Tissue specificity of *PregOBP56a* expression. cDNA was synthesized from total RNA extracted from each experimental tissue at day 0 adult stage. Gene expression was detected by RT-PCR. *actin* expression was used as an internal control. *PregOBP56a* was highly expressed in the oral disk. In contrast *CRLBP*, an *OBP* related to chemical reception, was expressed in the oral disk and antenna. B, Cellular localization of *PregOBP56a* expression in the oral disk by *in situ* hybridization. Sense and anti-sense probes were prepared by pSTP18 inserted with a PCR product amplified by primers PregOBP56a-1 and PregOBP56a-2. The left panel shows the localization of the oral disk within the head of a female. The right panel shows the localization of *PregOBP56a* transcripts in a cluster of cells that are localized between pseudotracheae. Scale bar is 20 µm.

To localize *PregOBP56a* expression in a female blowfly, RT-PCR analysis was carried out. We also investigated *CRLBP* expression as a reference of OBP related to chemical reception. Expression of *actin* as an internal control was detected from all experimental tissues. *PregOBP56a* was predominantly expressed in the oral disk with low level expression detected in the other chemosensory tissues including the maxillary palp and tarsus. On the other hand, *CRLBP* transcript was accumulated in the oral disk and antenna (Figure 3A). Oral disk-specific expression of *PregOBP56a* was consistent with the profile of proteins that was found by native PAGE analysis (Figs. 1 and 3A). To further localize *PregOBP56a* expression in the oral disk at the cellular level, *in situ* hybridization was performed. Using an anti-sense probe, *PregOBP56a* transcripts were detected in a cluster of cells localized between pseudotracheae. These cells were different from the accessory cells of the taste peg chemosensory sensillum, where OBPs are synthesized (Figure 3B). The control sense probe did not show any signals (data not shown). These results were identical to those found in a male adult blowfly (data not shown).

### Evaluation of expressed recombinant PregOBP56a

**Figure 4:**
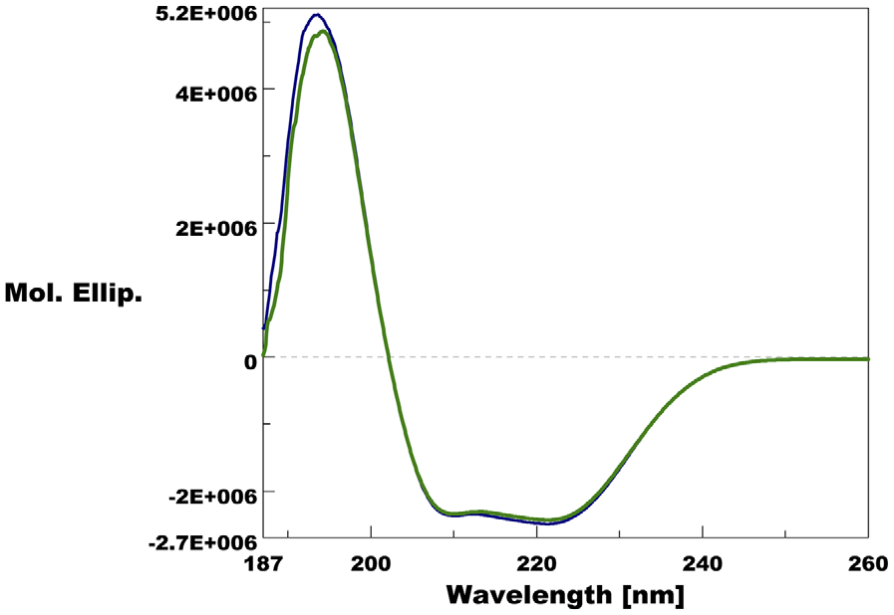
Far UV circular dichroism spectra of purified recombinant PregOBP56a. A positive peak of the CD spectra at 194 nm and two negative peaks at 209 and 221 nm are observed at pH 7 and pH 5, indicating that PregOBP56a is an α-helix-rich protein as other insect OBPs.

Identity and purity of the recombinant PregOBP56a was evaluated using Edman degradation, mass-finger printing, and MALDI TOF mass spectrometry. N-terminal sequence of the recombinant PregOBP56a was identical to that of the native protein (data not shown). Mass-finger printing analysis of the recombinant protein identified 7 peptide fragments (HLELTEEQK, VHFDECVK, NKDYANPTPAMK, CFGTCFFEK, DGVVQEAVVLEK, LSPHFGEEK, and IFECFEK), all of which were observed in amino acid sequence of mature PregOBP56a (Figure S1). The measured molecular mass of the recombinant protein was 13,245 Da (Figure S2), which was 6 Da less than the value calculated. These results suggested that the recombinant protein is a mature PregOBP56a that correctly formed 3 disulfide bridges as in the structure of insect classic OBPs. To analyze secondary structure of PregOBP56a, far-UV circular dichroism (CD) analysis was performed. The far-UV CD spectra showed one positive peak at 194 nm and two negative peaks at 209 nm and 221 nm (Figure 4), indicating that PregOBP56a is an α-helical-rich protein, as other insect OBPs reported previously [28], [30], [31], [32], [33], [34].

### Potential ligands of PregOBP56a expressing in the oral disk

Pseudotracheae on the oral disk of flies play a role in filtering out large particles in food [35], [36], [37], thus, blowflies only ingest liquid and/or tiny pieces of food. The chicken liver that is a primary component of the blowfly diet used for our colony contains hydrophobic long-chain fatty acids such as palmitic, stearic, oleic, and linoleic acids [38]. In order to address the ability of PregOBP56a to solubilize these fatty acids, we performed fluorescence competitive binding assay [27], [28].

**Figure 5:**
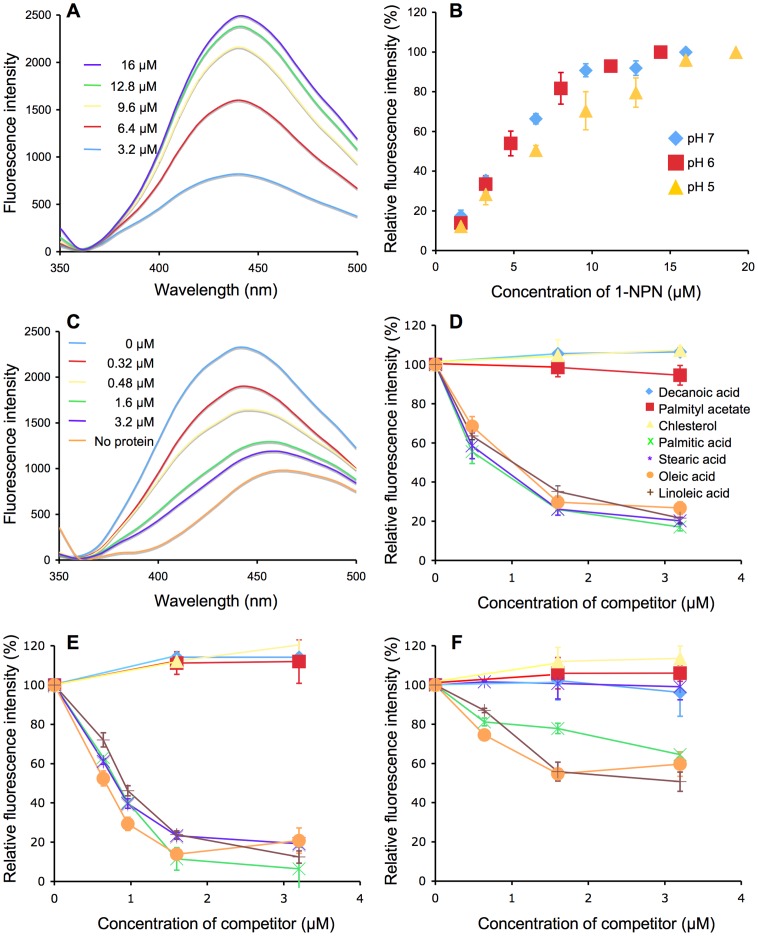
Binding of PregOBP56a to *N*-phenyl-1-naphthylamine (1-NPN) and potential ligands derived from chicken meat. A, A typical emission spectra following the addition of 1-NPN (3.2–16 µM final concentration) to recombinant PregOBP56a (10 µg/ml of 20 mM phosphate-citrate buffer, pH 7). The emission spectra were generated following excitation at a wavelength of 337 nm. B, Binding of 1-NPN to PregOBP56a at pH 7, 6, and 5. In these experiments PregOBP56a (10 µg/ml) was incubated with various concentrations (1.6–19.2 µM) of 1-NPN and emission at 440 nm was plotted. Values are means ± standard deviation, n = 3. C, Typical emission spectra following the addition of a competitor to PregOBP56a bound to 1-NPN. PregOBP56a (10 µg/ml) in 20 mM phosphate-citrate buffer at pH 7 was pre-incubated with 16 µM of 1-NPN (final concentration). Then, palmitic acid (0–3.2 µM final concentration) was added to the reaction, and the emission spectra (excitation a wavelength of 337 nm) were recorded. D, Binding of PregOBP56a to various ligands at pH 7. For each set of data, fluorescence values at a wavelength of 440 nm were plotted as percent of that obtained in the absence of the competitor. Values are means ± standard deviation, n = 3. The competitors are shown as insets. PregOBP56a bound palmitic, stearic, oleic, and linoleic acids but not decanoic acid, palmityl acetate, or cholesterol. E, Binding of PregOBP56a to various ligands at pH 6. The results were almost identical to those in panel D. F, Binding of PregOBP56a to various ligands at pH 5. The binding affinity of stearic acid decreased to same level as decanoic acid, palmityl acetate, and cholesterol. Due to low binding to PregOBP56a, the IC_50_ of other test competitors was not obtained.

Addition of the fluorescent reporter, *N*-phenyl-1-naphthylamine (1-NPN) to a solution of PregOBP56a gave fluorescence emission spectra with a peak at 440 nm (Figure 5A). The concentrations of 1-NPN that produced maximum fluorescence were 16, 14.4, and 19.2 µM at pH 7, 6, and 5, respectively (Figure 5B). The dissociation constants of PregOBP56a/1-NPN complex were 11.4, 12.3, and 17 µM at pH 7, 6, and 5, respectively.

The affinity of each ligand was evaluated using fluorescence competitive binding assay, where we measured the ability of each test compound to displace 1-NPN from the PregOBP56a/1-NPN complex. At pH 7, the addition of palmitic acid as a competitor to the PregOBP56a/1-NPN complex decreased the fluorescence intensity in a dose-dependent manner (Figure 5C). Also, at pH 7, a decrease in the fluorescence intensity (440 nm) was observed by addition of other fatty acids. Palmitic, stearic, oleic, and linoleic acids bound PregOBP56a with a calculated dissociation constant of 0.28, 0.35, 0.71, and 0.51 µM, respectively. However, the PregOBP56a did not bind to cholesterol (a major sterol in the chicken liver) and unrelated compounds such as decanoic acid (a short-chain fatty acid) or palmityl acetate (a long-chain ester) (Figure 5D). The binding assay results that were obtained at pH 6 were similar to those that were found at pH 7 (Figure 5E). At pH 6, palmitic, stearic, oleic, and linoleic acids bound PregOBP56a with calculated dissociation constants of 2.22, 1.24, 0.94, and 2.40 µM, respectively. On the other hand, at pH 5 the binding of PregOBP56a to these fatty acids was significantly decreased (Figure 5F). Even when the tested fatty acids were added at a final concentration of 16 µM, the fluorescence intensity did not decrease below 50% (data not shown) and the dissociation constants could not be calculated.

## Discussion

In blowflies the absorption of fatty acids from the meat diet is essential for normal progress of reproductive processes including JH synthesis, vitellogenesis, and mating behavior [8], [12], [39]. Although long-chain fatty acids are released from the triglyceride in meat by digestion of salivary lipase, these compounds are insoluble in an aqueous solution [13], [15]. In addition, since the pseudotrachea on the oral disk filter out particles, the blowflies only ingest a liquefied meal [35]. Therefore, some solubilizer is likely indispensable for the absorption of fatty acids.

PregOBP56a belongs to an insect classic OBP family. OBPs are known to accumulate in the sensillum cavity in chemosensory tissues and to encapsulate hydrophobic semiochemicals such as pheromones and odorants, and transfer then to the odorant receptor complex in the dendrite of the receptor neuron [17], [18], [19]. Pheromone-binding protein from the silkworm, *Bombyx mori* (BmorPBP) belonging to the same family is composed of 6 α-helices knitted with 3 disulfide bridges [40]. Because of the structural properties, this protein appears to be highly stable to endopeptidase digestion, heat inactivation, and low pH [26], [32]. Although crystallographic assignment is required, based on our results obtained by MALDI TOF mass spectrometry and circular dichroism analysis, PregOBP56a might have similar stability as BmorPBP.

A slight difference in the far-UV CD spectra of PregOBP56a was observed between pH 7 and pH 5 (Figure 4). A similar difference was previously observed in dipteran OBP [25]. These results suggested that the α-helical content of PregOBP56a might be unwound slightly, presumably causing dissociation of ligands at pH 5. It is known that mosquito OBPs form a dimer and that ligands bind in the long tunnel-binding cavity that is formed by dimer [41], [42], [43]. PregOBP56a possesses short peptides at the N- and C-termini, the structure of which is observed among dipteran OBPs but not in lepidopteran OBPs. Therefore PregOBP56a might bind fatty acids in a similar manner. Further analyses based on NMR spectroscopy and crystallography will clarify the molecular mechanism related to ligand binding and release.

In this study we demonstrated that PregOBP56a is expressed in a cluster of cells that is found between pseudotracheae. To date, OBPs are expressed not only in chemosensory tissues, but also in various parts of an insect body [28], [44], [45], [46], [47], [48], [49], [50]. It is reported that odorant-binding protein 19d from *Drosophila melanogaster* (DmelOBP19d) (previous name, PBPRP2) uniquely accumulates in the subcuticular space of the pseudotracheal region of the oral disk [47]. This localization resembles that of PregOBP56a. Dipteran insects might generally use this space to store OBPs. In the blowfly, it has been shown that the sensillar lymph containing CRLBP is exuded through a top pore of the taste sensillum on the oral disk [29], [51]. Similarly, PregOBP56a might be released through an unidentified pore(s) on the cuticle of oral disk or pseudotracheae. Further immunohistochemistry and electron microscopic study will clarify this secretion mechanism.

PregOBP56a showed specific binding to palmitic, stearic, oleic, and linoleic acids that are primary compounds found in chicken meat. This binding was highest, at pH 7 and pH 6 but decreased dramatically at pH 5. At 6 h postmortem the pH of the meat decreases to 6.1 by lactate generation during glycolysis [52]. PregOBP56a might be capable of binding palmitic, oleic, and linoleic acids in the meat up to 6 h postmortem. In addition, the pH of intestinal tract of the blowfly decreases from 7 (crop) to 5.5–6.2 (anterior midgut), and to 2–3.3 (coiled midgut) [53]. Thus, all fatty acids delivered by PregOBP56a will likely be dissociated in the midgut lumen. Subsequently, they can be absorbed and converted diacylglycerols in the enterocytes as is done in other insects [54]. Once in the hemolymph, lipophorin may take them to the target organs such as, corpus allata, fat bodies, and ovaries, respectively. We propose that PregOBP56a is a protein that solubilizes fatty acids during feeding and help in their delivery to the midgut for continuation of normal reproductive processes.

## Conclusions

Blowflies are economic pests of the wool industry and also insects of medical importance. Blowflies must feed on meat for normal progress of reproductive processes including juvenile hormone synthesis, vitellogenesis, and mating. The long-chain fatty acid components of meat are essential for this process. The newly identified PregOBP56a was specifically expressed in a cluster of the cells that are localized between pseudotracheae on the oral disk. These cells come into direct contact with the meat meal during feeding. PregOBP56a bound fatty acids at pH 7 and pH 6, but not at pH 5. These results suggested that PregOBP56a solubilizes fatty acids from fresh meat and delivers them to the midgut where low pH environment facilitates release and subsequent absorption. Since blowflies require long-chain fatty acids for normal reproductive processes, PregOBP56a might be a potential molecular target for controlling the reproduction of blowflies.

## Supporting Information

Figure S1 **Mass-finger printing of recombinant PregOBP56a.** A, Mass-fingerprinting of recombinant PregOBP56a. Molecular weight and corresponding positions were represented. Peaks with asterisks were [M+Na]^+^ions. B, Tryptic fragments of PregOBP56a. Identified fragments were represented in bold letters.
(PPT)

Figure S2 **MALDI TOF mass spectrometry of recombinant PregOBP56a.**
(PPT)

## References

[1] Hayashi T, Watanabe H, Watanabe M, Kobayashi M (2012) Mass occurrence of flies and seasonal changes in their species composition in the Tsunami disaster region after 2011 the Great East Japan Earthquake. Med Entomol Zool 63: 85–89.

[2] Knipling EF, Rainwater HT (1937) Species and incidence of dipterous larvae concerned in wound myiasis. J Parasitol 23: 451–455.

[3] Tellam RL, Bowles VM (1997) Control blowfly strike in sheep: current strategies and future prospects. Internatinal J Parasitol 27: 261–273. 10.1016/s0020-7519(96)00174-9 9138028

[4] Sawabe K, Hoshino K, Isawa H, Sasaki T, Kim KS, et al (2011) Blow flies were one of the possible candidates for transmission of highly pathogenic H5N1 avian influenza virus during the 2004 outbreaks in Japan. Influenza Res Treat 2011: 652652. 23074659 10.1155/2011/652652 PMC3447300

[5] Levot GW (1995) Resistance and the control of sheep ectoparasites. Int J Parasitol 25: 1355–1362. 8635885 10.1016/0020-7519(95)00070-i

[6] Zou B-X, Yin C-M, Stoffolano JG Jr, Tobe SS (1989) Juvenile hormone biosynthesis and release during oocyte development in *Phormia regina* Meigen. Physiol Entomol 14: 233–239.

[7] Liu M-A, Jones GL, Stoffolano Jr JG, Yin C-M (1988) Conditions for estimation of corpus allatum activity in the blowfly, *Phormia regina*, in vitro. Physiol Entomol 13: 69–79.

[8] Yin C-M, Qin W-H, Stoffolano JG Jr (1999) Regulation of mating behavior by nutrition and the corpus allatum in both male and female *Phormia regina* (Meigen). J Insect Physiol 45: 815–822. 12770294 10.1016/s0022-1910(99)00047-5

[9] Belles X, Martın D, Piulachs M-D (2005) The mevalonate pathway and the synthesis of juvenile hormone in insects. Annu Rev Entomol 50: 181–199. 15355237 10.1146/annurev.ento.50.071803.130356

[10] Stoffolano JG, Tobin EN, Wilson J, Yin C-M (1995) Diet affects insemination and sexual activity in male *Phormia regina* (Diptera: Calliphoridae). Ann Entomol Soc Am 88: 240–246.

[11] Orr CWM (1964) The influence of nutritional and hormonal factors on the chemistry of the fat body, blood, and ovaries of the blowfly *Phormia regina* Meig. J Insect Physiol 10: 103–119.

[12] Qin W-H, Yin C-M, Stoffolano Jr JG (1995) The role of the corpus allatum in the control of vitellogenesis and fat body hypertrophy in *Phormia regina* (Meigen). J Insect Physiol 41: 617–626.

[13] Kerlin RL, Hughes S (1992) Enzymes in saliva from four parastic arthropods. Med Vet Entomol 6: 121–126. 1330086 10.1111/j.1365-2915.1992.tb00587.x

[14] Hansen Bay CM (1978) Control of salivation in the blowfly *Calliphora* . J Exp Biol 75: 189–201.

[15] Vorum H, Brodersen R, Kragh-Hansen U, Pedersen AO (1992) Solubility of long-chain fatty acids in phosphate buffer at pH 7.4. Biochim Biophys Acta 1126: 135–142. 1627615 10.1016/0005-2760(92)90283-2

[16] Ghafouri B, Tagesson C, Lindahl M (2003) Mapping of proteins in human saliva using two-dimensional gel electrophoresis and peptide mass fingerprinting. Proteomics 3: 1003–1015. 12833525 10.1002/pmic.200300426

[17] Vogt RG (2003) Biochemical diversity of odor detection: OBPs, ODEs and SNMPs. In: Blomquist G, Vogt RG, editors. Insect pheromone biochemistry and molecular biology: The biosynthesis and detectionof pheromones and plant volatiles. San Diego: Elsevier Academic Press. pp. 391–445.

[18] Leal WS (2003) Protein that make sense. In: Blomquist GJ, Vogt RG, editors. Insect pheromone biochemistry and molecular biology: The biosynthesis and detection of pheromones and plant volatiles. San Diego: Elsevier Academic Press. pp. 447–476.

[19] Pelosi P, Zhou J-J, Ban LP, Calvello M (2006) Soluble proteins in insect chemical communication. Cell Mol Life Sci 63: 1658–1676. 16786224 10.1007/s00018-005-5607-0 PMC11136032

[20] Nishimura T, Seto A, Nakamura K, Miyama M, Nagao T, et al (2005) Experiential effects of appetitive and nonappetitive odors on feeding behavior in the blowfly, *Phormia regina*: a putative role for tyramine in appetite regulation. J Neurosci 25: 7507–7516. 16107638 10.1523/JNEUROSCI.1862-05.2005 PMC6725415

[21] Tamura K, Peterson D, Peterson N, Stecher G, Nei M, et al (2011) MEGA5: Molecular evolutionary genetics analysis using maximum likelihood, evolutionary distance, and maximum parsimony methods. Mol Biol and Evol 28: 2731–2739. 21546353 10.1093/molbev/msr121 PMC3203626

[22] Ishida Y, Leal WS (2002) Cloning of putative odorant-degrading enzyme and integumental esterase cDNAs from the wild silkmoth, *Antheraea polyphemus* . Insect Biochem Mol Biol 32: 1775–1780. 12429129 10.1016/s0965-1748(02)00136-4

[23] Vosshall LB, Amrein H, Morzov PS, Rzhetsky A, Axel R (1999) A spatial map of olfactory receptor expression in the *Drosophila* antenna. Cell 96: 725–736. 10089887 10.1016/s0092-8674(00)80582-6

[24] Ishida Y, Ozaki M (2011) A putative octopamine/tyramine receptor mediating appetite in a hungry fly. Naturwissenschaften 98: 635–638. 21603928 10.1007/s00114-011-0806-z

[25] Leal WS, Barbosa RMR, Xu W, Ishida Y, Syed Z, et al (2008) Reverse and conventional chemical ecology approaches for the development of oviposition attractants for *Culex* mosquitoes. PLoS ONE 3: e3045. 18725946 10.1371/journal.pone.0003045 PMC2516325

[26] Wojtasek H, Leal WS (1999) Conformational change in the pheromone-binding protein from *Bombyx mori* induced by pH and by interaction with membranes. J Biol Chem 274: 30950–30956. 10521490 10.1074/jbc.274.43.30950

[27] Ban L, Scaloni A, Brandazza A, Angeli S, Zhang L, et al (2003) Chemosensory proteins of *Locusta migratoria* . Insect Mol Biol 12: 125–134. 12653934 10.1046/j.1365-2583.2003.00394.x

[28] Xu W, Cornel AJ, Leal WS (2010) Odorant-binding proteins of the malaria mosquito *Anopheles funestus sensu stricto* . PLoS ONE 5: e15403. 21042539 10.1371/journal.pone.0015403 PMC2962654

[29] Ozaki M, Morisaki K, Idei W, Ozaki K, Tokunaga F (1995) A putative lipophilic stimulant carrier protein commonly found in the taste and olfactory systems: A unique member of the pheromone-binding protein superfamily. Eur J Biochem 230: 298–308. 7601113 10.1111/j.1432-1033.1995.0298i.x

[30] Lartigue A, Gruez A, Briand L, Blon F, Bezirard V, et al (2004) Sulfur single-wavelength anomalous diffraction crystal structure of a pheromone-binding protein from the honeybee Apis mellifera L. J Biol Chem 279: 4459–4464. 14594955 10.1074/jbc.M311212200

[31] Lartigue A, Gruez A, Spinelli S, Riviere S, Brossut R, et al (2003) The crystal structure of a cockroach pheromone-binding protein suggests a new ligand binding and release mechanism. J Biol Chem 278: 30213–30218. 12766173 10.1074/jbc.M304688200

[32] Leal WS, Nikonova L, Guihong P (1999) Disulfide structure of the pheromone binding protein from the silkworm moth, *Bombyx mori* . FEBS Letters 464: 85–90. 10611489 10.1016/s0014-5793(99)01683-x

[33] Scaloni A, Monti M, Angeli S, Pelosi P (1999) Structural analysis and disulfide-bridge pairing of two odorant-binding proteins from *Bombyx mori* . Biochem Biophys Res Commun 266: 386–391. 10600513 10.1006/bbrc.1999.1791

[34] Vandermoten S, Francis F, Haubruge E, Leal WS (2011) Conserved odorant-binding proteins from aphids and eavesdropping predators. PLoS ONE 6: e23608. 21912599 10.1371/journal.pone.0023608 PMC3160308

[35] Graham-Smith GS (1911) Some observations on the anatomy and function of the oral sucker of the blowfly (*Calliphora erythrocephala*). J Hyg 11: 390–408.399. 20474464 10.1017/s0022172400016843 PMC2167262

[36] Coronado-Gonzalez PA, Vijaysegaran S, Robinson AS (2008) Functional morphology of the mouthparts of the adult Mediterranean fruit fly, *Ceratitis capitata* . J Insect Sci 8.73

[37] Sukontason K, Methanitikorn R, Kurahashi H, Vogtsberger RC, Sukontason KL (2008) External morphology of *Chrysomya pinguis* (Walker) (Diptera: Calliphoridae) revealed by scanning electron microscopy. Micron 39: 190–197. 17339116 10.1016/j.micron.2007.01.004

[38] Almeida JCd, Perassolo MS, Camargo JL, Bragagnolo N, Gross JL (2006) Fatty acid composition and cholesterol content of beef and chicken meat in Southern Brazil. RBCS 42: 109–117.

[39] Orr CWM (1964) The influence of nutritional and hormonal factors on egg development in the blowfly, *Phormia regina* (Meig.). J Insect Physiol 10: 53–64.

[40] Sandler BH, Nikonova L, Leal WS, Clardy J (2000) Sexual attraction in the silkworm moth: structure of the pheromone-binding-protein-bombykol complex. Chem Biol 7: 143–151. 10662696 10.1016/s1074-5521(00)00078-8

[41] Tsitsanou KE, Thireou T, Drakou CE, Koussis K, Keramioti MV, et al (2011) *Anopheles gambiae* odorant binding protein crystal complex with the synthetic repellent DEET: implications for structure-based design of novel mosquito repellents. Cell. Mol. Life Sci 69: 283–297. 21671117 10.1007/s00018-011-0745-z PMC11114729

[42] Wogulis M, Morgan T, Ishida Y, Leal WS, Wilson DK (2006) The crystal structure of an odorant binding protein from *Anopheles gambiae*: Evidence for a common ligand release mechanism. Biochem Biophys Res Commun 339: 157–164. 16300742 10.1016/j.bbrc.2005.10.191

[43] Mao Y, Xu X, Xu W, Ishida Y, Leal WS, et al (2010) Crystal and solution structures of an odorant-binding protein from the southern house mosquito complexed with an oviposition pheromone. Proc Natl Acad Sci USA 107: 19102–19107. 20956299 10.1073/pnas.1012274107 PMC2973904

[44] Hekmat-Scafe DS, Dorit RL, Carlson JR (2000) Molecular evolution of odorant-binding protein genes *OS-E* and *OS-F* in Drosophila. Genetics 155: 117–127. 10790388 10.1093/genetics/155.1.117 PMC1461081

[45] Galindo K, Smith DP (2001) A large family of divergent drosophila odorant-binding proteins expressed in gustatory and olfactory sensilla. Genetics 159: 1059–1072. 11729153 10.1093/genetics/159.3.1059 PMC1461854

[46] Pelletier J, Leal WS (2009) Genome analysis and expression patterns of odorant-binding proteins from the southern house mosquito *Culex pipiens quinquefasciatus* . PLoS ONE 4: e6237. 19606229 10.1371/journal.pone.0006237 PMC2707629

[47] Shanbhag SR, Park S-K, Pikielny CW, Steinbrecht RA (2001) Gustatory organs of *Drosophila melanogaster*: fine strucutre and expression of the putative odorant-binding protein PBPRP2. Cell Tissue Res 304: 423–437. 11456419 10.1007/s004410100388

[48] Biessmann H, Nguyen QK, Le D, Walter MF (2005) Microarray-based survey of a subset of putative olfactory genes in the mosquito *Anopheles gambiae* . Insect Mol Biol 14: 575–589. 16313558 10.1111/j.1365-2583.2005.00590.x

[49] Park S-K, Shanbhag SR, Wang Q, Hasan G, Steinbrecht RA, et al (2000) Expression patterns of two putative odorant-binding proteins in the olfactory organs of *Drosophila melanogaster* have different implications for their functions. Cell Tissue Res 300: 181–192. 10805087 10.1007/s004410000187

[50] Pikielny CW, Hasan G, Rouyer F, Rosbash M (1994) Members of a family of Drosophila putative odorant-binding proteins are expressed in different subsets of olfactory hairs. Neuron 12: 35–49. 7545907 10.1016/0896-6273(94)90150-3

[51] Dethier VG (1976) The hungry fly. Cambridge: Harvard University Press. 512 p.

[52] Savenije B, Lambooij E, Gerritzen MA, Venema K, Korf J (2002) Efects of feed deprivation and transport on preslaughter blood metabolites, early postmortem muscle metabolites, and meat quality. Poult Sci 81: 699–708. 12033421 10.1093/ps/81.5.699

[53] Taylor CW (1985) Calcium regulation in blowflies: absence of a role for midgut. Am J Physiol 249: R209–R213. 4025578 10.1152/ajpregu.1985.249.2.R209

[54] Arrese EL, Canavoso LE, Jouni ZE, Pennigton JE, Tsuchida K, et al (2001) Lipid storage and mobilization in insects: current status and future directions. Insect Biochem Mol Biol 31: 7–17. 11102830 10.1016/s0965-1748(00)00102-8

