# Supplementary material for: Fatty Acid Solubilizer from the Oral Disk of the Blowfly
Source: PLoS One. 2013 Jan 11;8(1):e51779. doi: 10.1371/journal.pone.0051779 (PMC3543412; doi:10.1371/journal.pone.0051779)
Supplement: Figure S1 — Mass-finger printing of recombinant PregOBP56a. A, Mass-fingerprinting of recombinant PregOBP56a. Molecular weight and corresponding positions were represented. Peaks with asterisks were [M+Na]+ions. B, Tryptic fragments of PregOBP56a. Identified fragments were represented in bold letters. (PPT) [file pone.0051779.s001.ppt]

## Slide 1
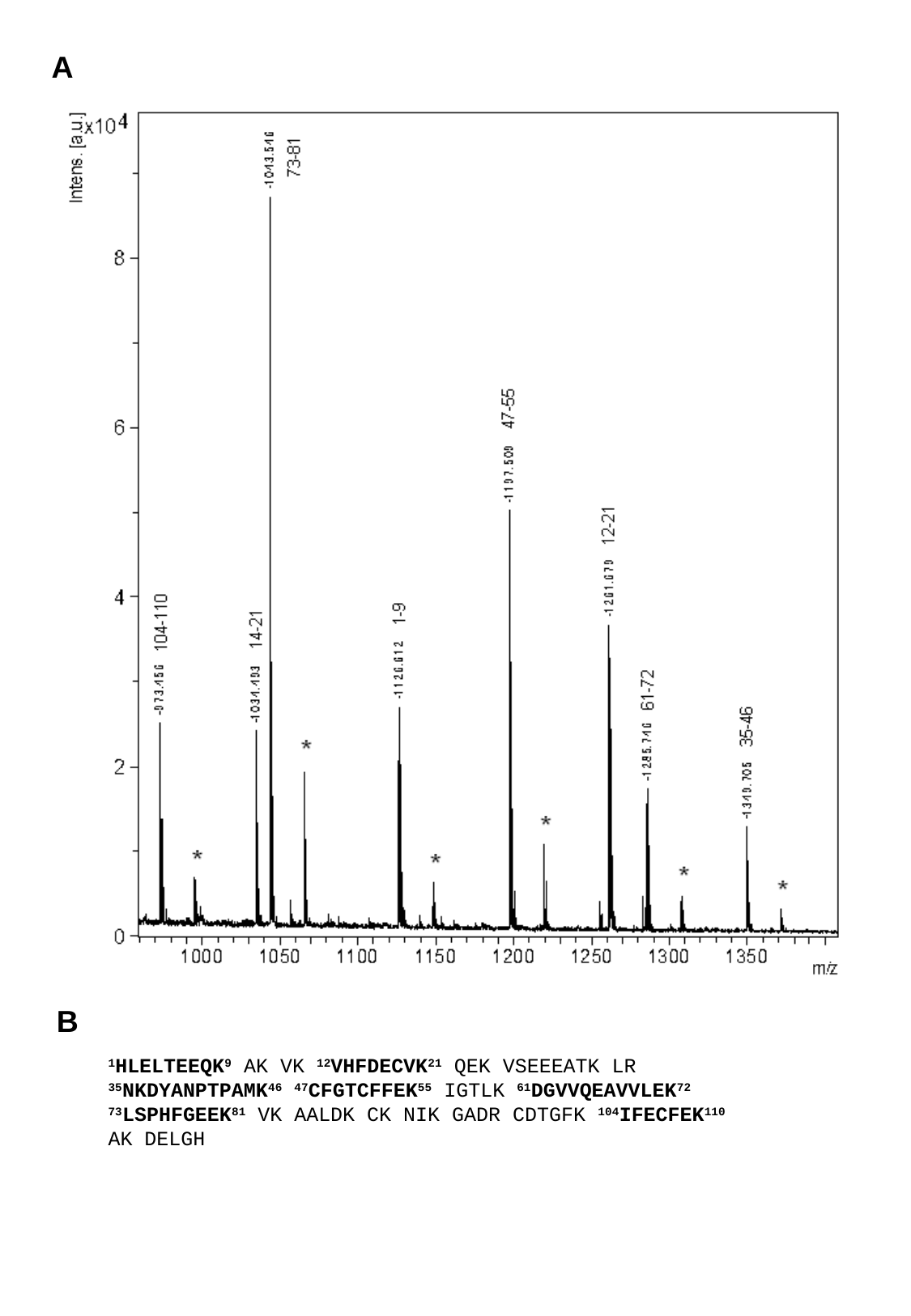

A
B
1HLELTEEQK9 AK VK 12VHFDECVK21 QEK VSEEEATK LR 35NKDYANPTPAMK46 47CFGTCFFEK55 IGTLK 61DGVVQEAVVLEK72 73LSPHFGEEK81 VK AALDK CK NIK GADR CDTGFK 104IFECFEK110 AK DELGH
