# Supplementary figures and images for: Fatty Acid Solubilizer from the Oral Disk of the Blowfly
Source: PLoS One. 2013 Jan 11;8(1):e51779. doi: 10.1371/journal.pone.0051779 (PMC3543412; doi:10.1371/journal.pone.0051779)

## Slide 1
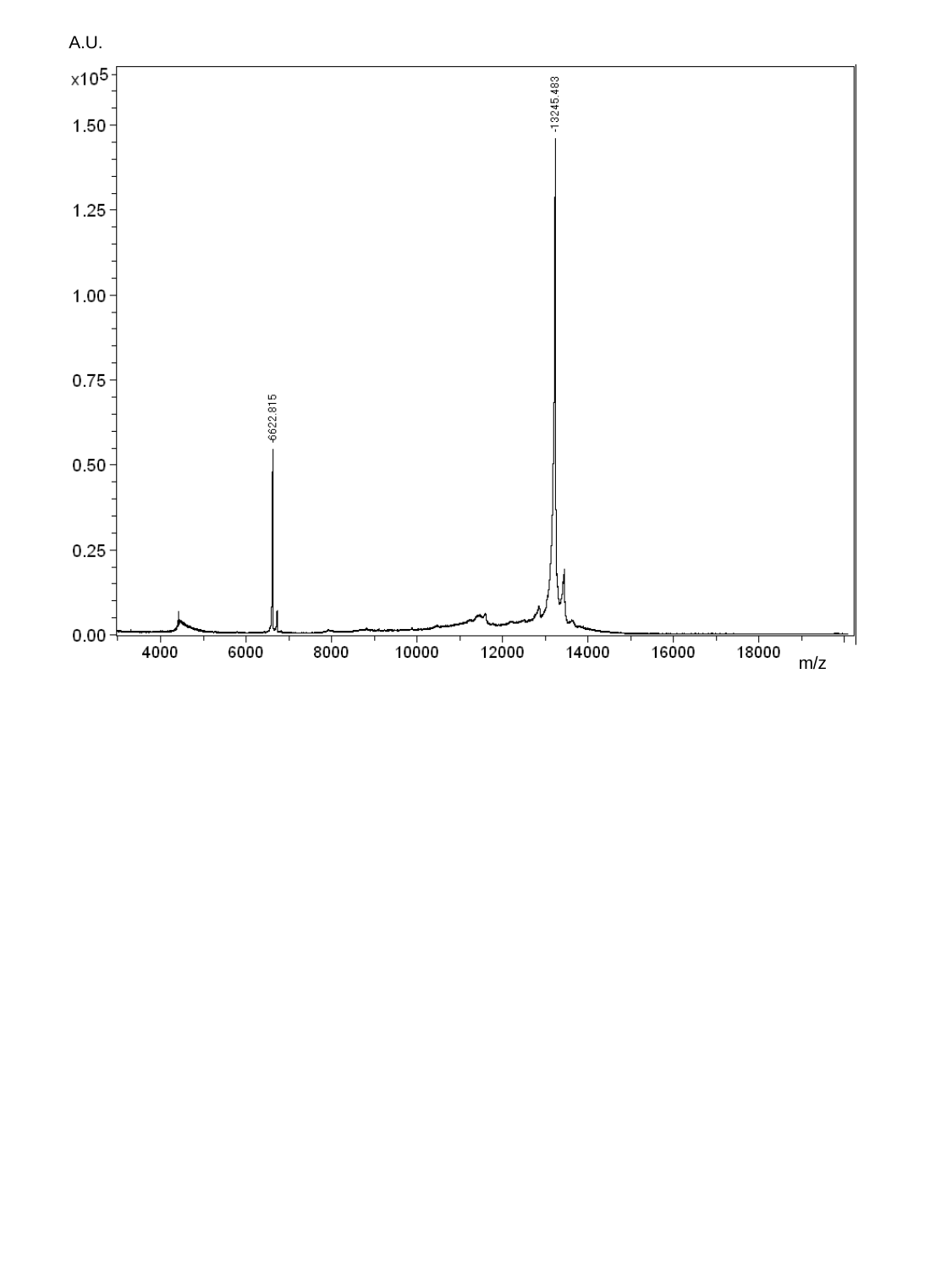

A.U.
m/z

Supplement: Figure S2 — MALDI TOF mass spectrometry of recombinant PregOBP56a. (PPT) [file pone.0051779.s002.ppt]
